# Mechanical Behavior Modelling and Filler Geometry Effect of Glass Filler Reinforced Starch-Epoxy Hybrid Matrix Composites

**DOI:** 10.3390/ma14216587

**Published:** 2021-11-02

**Authors:** Lykourgos C. Kontaxis, Foteini K. Kozaniti, George C. Papanicolaou

**Affiliations:** Composite Materials Group, Department of Mechanical Engineering and Aeronautics, University of Patras, GR 265 04 Patras, Greece; fkozaniti@upatras.gr (F.K.K.); gpapan@upatras.gr (G.C.P.)

**Keywords:** starch, epoxy, glass fillers, hybrid polymer matrix, flexural modulus, flexural strength, degree of adhesion, degree of dispersion, filler aspect ratio, prediction model

## Abstract

The aim of the present study is to investigate the inclusion geometry and concentration effect on the quasi-static properties of a starch-epoxy hybrid matrix composite. The composites investigated consisted of a starch-epoxy hybrid matrix reinforced with four different glass inclusions such as 3 mm long chopped strands, 0.2 mm long short glass fibers, glass beads (120 μm in diameter) and glass bubbles (65 μm in diameter) at different concentrations. The flexural modulus and the strength of all materials tested were determined using three-point bending tests. The Property Prediction Model (PPM) was applied to predict the experimental findings. The model predicted remarkably well the mechanical behavior of all the materials manufactured and tested. The maximum value of the flexural modulus in the case of the 3 mm long chopped strands was found to be 75% greater than the modulus of the hybrid matrix. Furthermore, adding glass beads in the hybrid matrix led to a simultaneous increase in both the flexural modulus and the strength.

## 1. Introduction

Progress in the science and technology of polymers has permitted the extended use and production of polymers and especially of composite materials. However, issues such as recycling and degradation require further improvements; therefore, a further investigation of the materials from natural resources has been suggested.

The current tendency is for the large-scale production of bio-based polymers. According to the statistics, 75% of the produced starch polymers are used in packaging. Nowadays, another area of application for modified starch polymers is in the transportation sector [1]. As far as this area is concerned, the advantages of starch include a lower rolling resistance, noise reduction, reduced fuel consumption and CO_2_ emissions, and reduced manufacturing energy requirements. Because of their relatively low cost, polymers based on starch are an attractive alternative to polymers based on petrochemicals. Generally, starch is added to improve biodegradation or to decrease the cost of the final plastic product [2,3,4,5,6,7]. For instance, Bulatovic et al. [6] manufactured polylactic acid (PLA)/polycaprolactone (PCL)/thermoplastic starch (TPS) polymer blends with compositional variations, while Bai et al. [7] manufactured Poly (butylene adipate-co-terephthalate) (PBAT)/TPS blends and both concluded that besides being an environmentally friendly material, the addition of TPS reduces the material’s cost significantly.

Corn starch was selected as a natural material while granular starch has been used as filler in some types of plastics since 1970s. According to the literature [8,9,10,11,12,13,14,15], corn starch can improve both the mechanical strength and stiffness of polymers. More precisely, as the starch content is increased, both the modulus and strength increase up to a certain maximum value of starch concentration, beyond which a reduction in the modulus and strength is usually observed due to agglomerations of starch granules. Willett and Felker [8] documented an increase in the yield strength in the case of starch-filled poly (ester amide) materials, while Ogunsona et al. [10] stated that some of the properties that can be achieved via starch modification include thermal stability, amphiphilicity, paste clarity, mechanical strength, freeze-thaw stability, and retrogradation resistances amongst others. Furthermore, Nawang et al. [11] incorporated sago starch into LLDPE by melt-mixing and found that the optimum volume filler content was 15%, above which a sharp drop in the mechanical properties occurred.

In the present investigation, fibrous and/or granular glass fillers were added for further reinforcement of the hybrid starch-epoxy matrix. Glass fillers can be classified by their shape as platelets (e.g., talc), fibrous and spherical, or glass microspheres. According to their synthesis, various types of glasses are available, such as borosilicate, soda-lime, and E-Glass. Amongst them, the most common type is the E-Glass. E-Glass is characterized by high strength and elasticity modulus values and good electrical properties, while it is not affected by weather conditions. In addition, E-Glass can be modified into useful fibers [16]. In this study, E-Glass was used in the form of chopped glass fibers, soda-lime solid glass beads, and borosilicate hollow glass spheres.

Glass microspheres, which can be hollow or solid, are spherical particles that have a typical diameter range from 1 to 1000 μm. The low density of hollow glass spheres can reduce the weight of the composite material. If they are used as fillers, the modulus can be increased, but not the tensile/flexural/impact strength. Moreover, the solid glass beads are spherical, smooth, and hard. Therefore, they can withstand high compression loads and the round shape also helps by spreading the stress [17,18,19].

Furthermore, chopped fibers are another commonly used geometry of glass fillers. Continuous rovings can be chopped and thus fibers of 0.1–10 mm in length can be manufactured. Improved strength and stiffness are undoubtedly the main characteristics of composites reinforced with short fibers. In the case of unreinforced composites, other important properties, such as toughness, are decreased compared to those of reinforced composites. In addition, flexibility in processing is one of the advantages of short fiber reinforced composites [20,21].

As stated by Yesgat et al. [22], most studies on mechanical characterization, and in particular on the fracture mechanics of particulate composites, are conducted either by reinforcing with regular shaped (mostly spherical) particles or by embedding randomly shaped fillers into the matrix material [23,24,25]. However, there are far fewer studies on the effect of the filler geometry of the same material on the mechanical properties of a polymer composite [26,27,28,29,30]. The results on the mechanical behavior of composite materials filled with different geometries/shapes of the same material in the scientific community are few and sometimes contradictory. Kushvaha et al. [27] concluded that the rod-shaped fillers produce the highest crack initiation toughness as well post-initiation KI values followed by flakes and spheres, respectively, while Malucelli et al. [29] did not discover a discernable difference in the dynamic properties of his ZnO filled composites with respect to filler geometry. Singh et al. [26] reported that spherical particles had a negligible influence on the stress vs. strain curves of composites, attributed to the large inter-particle separation distance, while the mechanical behavior of composites is considerably affected by milled-fibers due to the relatively larger surface area to volume ratio of fillers.

Finally, Leluk et al. [30] concluded that the aspect ratio of an introduced filler plays a key role in the materials’ end properties. In addition, Bek et al. [31] stated that as the aspect ratio increases, the friction between the polymer matrix and the filler particles increases, which inhibits the movement of polymeric chains and results in a higher viscosity. Even thermal properties are affected by geometry and the aspect ratio. As stated by Zhao et al. [32], in order to improve the thermal conductivity of Aluminosilicate- and Aluminum Oxide-Filled Thermosets, fillers with cuboid geometry are more effective than those with sphere geometry. This is attributed to the smaller surface area of sphere fillers and the more thermal-resistant interfaces, while Cho et al. [33] observed that the composites with S-Carbon nanofibers (average *L*/*d* = 10) show better thermal stability than one of the composites with L- Carbon nanofibers (average *L*/*d* = 70).

Taking into consideration all the previous information and the fact that few researchers had dealt with untreated starch, Papanicolaou et al. investigated the quasi-static and viscoelastic behavior of untreated starch reinforced with epoxy resin composites [12]. In addition, according to the authors of [34], when an epoxy resin-modified starch sizing agent is used, the mechanical properties of glass fiber reinforced epoxy composites including the tensile, bending, and impact strength at break are all greatly improved.

As a sequel to our previous studies, the percentage of the untreated starch in the epoxy resin was kept constant and equal to the optimum one already found in our previous publication (10 wt %). Then, the hybrid matrix produced was further reinforced with glass fillers of various shapes and concentrations. All composites were mechanically characterized, and their mechanical behavior was predicted using the Property Prediction Model (PPM), a model previously developed by the third author. It was found that the predicted values were in good agreement with the experimental results.

## 2. Materials and Methods

### 2.1. Materials

Normal corn starch was purchased from AKIS Corp (Aspropyrgos, Greece), which is a local company. The physical properties of corn starch are given in Table 1, as taken from the literature [35]. The epoxy resin applied was RenLam CY219 (Bisphenol A) combined with a curing agent HY 5161 (amine) at a 2:1 ratio by weight. Gelling time was 24 h at 50 °C, and the density of the cured polymer was 1.1 g/cm^−3^. The viscosity of the systems CY219 and HY 5161 was 1–1.2 Pas at 25 °C. The supplier of the glass fillers was R&G GmbH (Waldenbuch, Germany). Chopped Glass Fiber strands 3 mm in length, Chopped Glass Fiber strands 0.2 mm in length, Glass Microspheres (120 μm ф) and Glass Bubbles K1 (65 μm ф) were used. Their properties are presented on Table 2, as given by the manufacturers.

### 2.2. Manufacture of the Specimens

The resin was placed in the oven for 10 min at 40 °C to decrease its viscosity. Likewise, starch was placed in an oven at 50 °C for 24 h to remove humidity. The starch (10 wt %) and the glass filler were mechanically mixed before being added to the resin. This was important in order to achieve a uniform distribution of the fillers into the matrix. The polymer resin and the starch/glass particles mixture were carefully mixed by an electrical stirrer for 10 min in proper quantities (see Table 3), to achieve a uniform distribution of the fillers into the matrix. Then, the hardener was added to the blend and was mechanically mixed for 5 min. Afterwards, the mixture was placed in a vacuum chamber for 5–6 min to reduce the amount of entrapped air. The final product was then poured in a proper metallic mold and cured in an oven at 50 °C for 24 h under atmospheric pressure. The specimens were removed from the mold and post-cured in an oven at 50 °C for 2 h. The final specimens had a total length of 100 mm, a total width of 12.8 mm, a total depth of 2.5 mm, and a total gauge length of 63 mm. It should be mentioned that, in the case of glass bubbles and the 3 mm long glass fibers, close molds were used, and they were fully rotated every 10 min for the first hour in order to prevent the agglomeration of the fillers at the edges of the specimens since glass bubbles would float on the surface, while the 3 mm long glass fibers would sink to the bottom otherwise.

### 2.3. Quasi-Static Mechanical Tests

Three-point bending tests were carried out according to ASTM D 0790 by means of a conventional universal testing machine (INSTRON 430, High Wycombe, UK), at room temperature. All specimens had the dimensions 100 × 12.8 × 2.5 mm^3^ and a span length of 63 mm, while in all cases a constant crosshead speed of 1 mm/min was applied. Five or more specimens were manufactured and tested per each individual composition and inclusion type studied, to ensure the repeatability of the results.

### 2.4. Microstructural Analysis of the Composites

The morphology of the microstructure of the specimens manufactured was analyzed by means of a Scanning Electron Microscope (SEM). The SEM used was model QUANTA FEG 250 produced by Thermo Fisher Scientific (Waltham, MA, USA).

The void content of the specimens manufactured for the three-point bending tests was determined according to ASTM D 2734. The density of the specimens was calculated in accordance with ASTM D 1505.

## 3. Theoretical Background

### 3.1. Property Prediction Model

The model was developed by G.C. Papanicolaou [40,41,42] in order to describe/predict the variation of any physical/mechanical property (*P_c_*) of a composite material with filler loading (*C_f_*) and/or with any other parameter affecting the overall behavior of the material. At this point, it must be stressed that for the application of the model, two experimental points only are needed. The first one (*C*_1_, *P*_1_) should be selected at a very low filler concentration where the dominant factor affecting the composite behavior is filler-matrix adhesion, while the second one (*C*_2_, *P*_2_), should be selected at the maximum possible filler concentration where the filler dispersion is the dominant parameter affecting the overall composite behavior.

In addition, for the model development, the following assumptions were made:

1. The model considers that, for any conditions given, the main parameters affecting the composite behavior are the filler-matrix adhesion and the filler dispersion within the matrix.
2. The change of any property of the composite with the inclusion content can be described by a second-degree polynomial, which, depending on the value of its coefficients, can represent a straight line and/or a concave or convex parabolic curve:
  (1) $$P_{c}=AC_{f}^{2}+BC_{f}+P_{m},$$
  where, *P_m_* is the property of the matrix.

Having two experimental points, (*C*_1_*, P*_1_) and (*C*_2_, *P*_2_), then by setting:

(2) $$A=\frac{P_{2}-P_{m}}{C_{2}\left(C_{2}-C_{1}\right)}-\frac{P_{1}-P_{m}}{C_{1}\left(C_{2}-C_{1}\right)}$$

and

(3) $$B=\frac{\left(P_{1}-P_{m}\right)C_{2}}{C_{1}\left(C_{2}-C_{1}\right)}-\frac{\left(P_{2}-P_{m}\right)C_{1}}{C_{2}\left(C_{2}-C_{1}\right)}$$

Equation (1) can be solved for the composite property.

### 3.2. Degree of Adhesion and Degree of Dispersion

**Figure 1:**
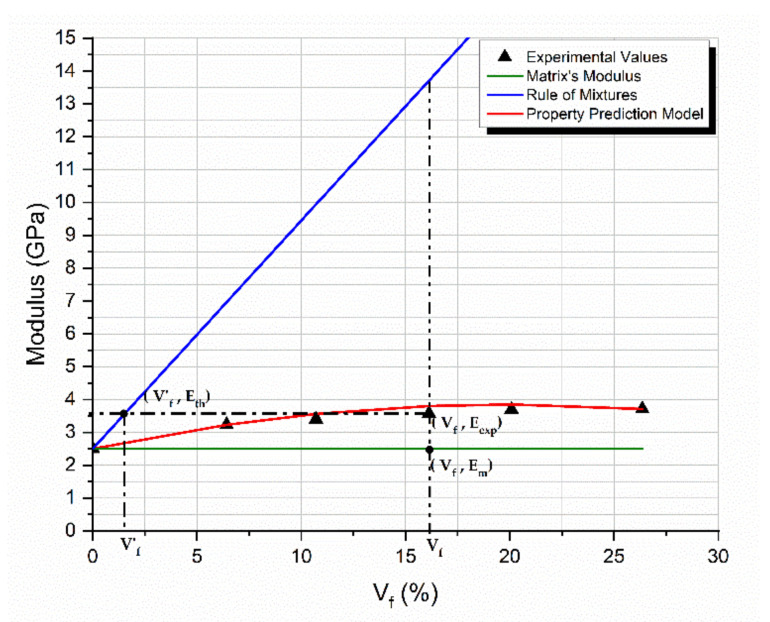
Experimental values of the stiffness variation with the filler-volume fraction of a particulate composite, along with the matrix modulus, *E_m_*, the rule of mixtures prediction, *E_th_*, and the respective PPM prediction.

For a better understanding of the PPM model, let us assume the experimental curve for the stiffness variation with the filler-volume fraction of a particulate composite as shown in Figure 1. In the same figure, the matrix modulus, *E_m_*, and the rule of mixtures prediction, *E_th_*, as well as the respective PPM prediction, *E_pred_* are also depicted.

The rule of mixtures is expressed by the equation:

(4) $$E_{th}=E_{c}=E_{f}V_{f}+E_{m}(1-V_{f})$$

It is an ideal rule, representing the stiffness values for perfect filler-matrix adhesion (100% = 1). However, as one can easily observe, there is a great difference between the experimental values and the respective ideal values. The observed difference in stiffness is mainly due to the combined effect of both adhesion and dispersion. This difference in stiffness values is reflected to the filler-volume fraction shift $V_{f}^{\prime }-V_{f}$ which represents the additional filler loading needed to achieve the same stiffness value for the real composite with that of the ideal one. Taking now the triangles similarities we can observe that:

(5) $$n=\frac{V_{f}-{V^{\prime }}_{f}}{V_{f}}=\frac{E_{th}-E_{\exp}}{E_{th}-E_{m}}$$

This equation states that for:

(6) $$\begin{array}{l} E_{\exp}=E_{th}\Rightarrow \;n=0\\ E_{\exp}=E_{m}\Rightarrow \;n=1 \end{array}$$

Using now the predicted values, *E_pred_*, which are too close to the experimental ones, Equation (5) can be written as:

(7) $$n=\frac{V_{f}-{V^{\prime }}_{f}}{V_{f}}=\frac{E_{th}-E_{pred}}{E_{th}-E_{m}}$$

from which we can obtain the modulus predicted (or experimental) values, as:

(8) $$E_{\exp}=E_{pred}=(1-n)E_{th}+nE_{m}$$

Parameter *n* is a parameter affected by both the adhesion (y-axes differences) and dispersion (x-axes differences). This parameter should be included in the expression for the definition of both the degree of adhesion, *K*, and that of the degree of dispersion, *L*. In addition, both parameters should vary within the interval 1 to 0, where 1 corresponds to the perfect and 0 to the worst conditions. Finally, it must be recognized that adhesion and dispersion are two antagonistic phenomena with *K* affecting the composite modulus and strength through the load to strain transfer fraction from the matrix to the inclusion. In addition, the degree of dispersion, *L*, affects the composite mechanical properties through the filler-matrix contact area, which in turn depends on the filler dispersion within the matrix and the development of filler aggregates with increasing *V_f_*.

Such expressions for *K* and *L* that comply with the above-mentioned conditions, are:

(9) $$K=\left(1-n\right)V_{f}^{n}$$

and

(10) $$L=n{\left(1-V_{f}\right)}^{n}$$

From the above equations we observe that:

(11) $$\begin{array}{l} E_{\exp}=E_{th}\Rightarrow \;n=0\;\Rightarrow \;K=1,\;L=0\\ E_{\exp}=E_{m}\Rightarrow \;n=1\;\Rightarrow {\;K=0,\;L=1-V}_{f} \end{array}$$

Equation (11) gives a deeper physical meaning of both parameters *K* and *L*. Both parameters vary with the filler-volume fraction, since by adding more filler into the matrix material, the effective filler-matrix contact area varies in a complex manner due to the agglomerations that might be created, rendering, at the same time, the filler dispersion within the matrix non-perfect. It should be noted that both the *K* and *L* parameters are defined uniquely by the stiffness variation and not by any other property variation as predicted by PPM. This is because the modulus is the only reliable variable as compared with other properties such as strength or strain at failure, since the parameters related to the ultimate properties, amongst others, are also affected by void content and stress-singularity sites developed within the composite during manufacturing, leading to loss of repeatability of the experimental results.

Now, taking into consideration the *K* and *L* definitions, Equation (8) can be written as:

(12) $$E_{pred}=\frac{K}{V_{f}^{n}}E_{th}+\frac{L}{{\left(1-V_{f}\right)}^{n}}E_{m}$$

### 3.3. Model Application

In this sub-chapter a step-by-step methodology is proposed for applying the PPM model on our experimental data.

- **Step 1.** Given two experimental points, (*C*_1_, *P*_1_) at low *C_f_* and (*C*_2_, *P*_2_) at high *C_f_*, the modulus prediction is obtained.
- **Step 2.** Using the modulus predicted values and applying Equations (7) and (9), the degree of adhesion *K* variation with the filler-volume fraction, *C_f_* is obtained.
- **Step 3.** Using the modulus predicted values and applying Equations (7) and (10), the degree of dispersion *L* variation with the filler-volume fraction *C_f_* is obtained.

## 4. Results and Discussion

In the present section, the experimental findings, and the respective PPM predictions regarding the flexural modulus and strength of all types of the composites manufactured as a function of the filler-volume fraction are presented. They are divided according to the filler type of the composite examined. In addition, in each case, the respective results concerning the degree of adhesion and dispersion variation with the filler-volume fraction are presented and discussed.

### 4.1. Glass Spheres

**Figure 2:**
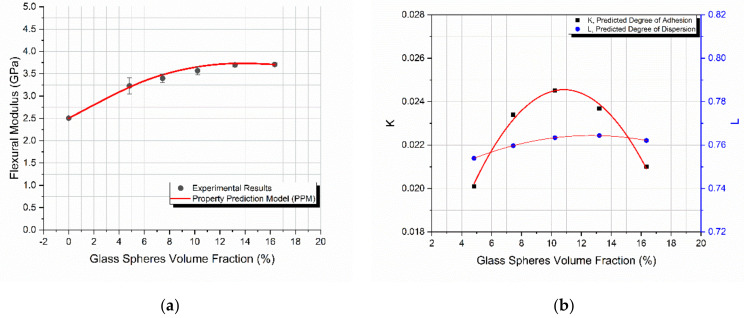
Glass spheres starch-epoxy composites variation with the filler-volume fraction *V_f_*, of (**a**) flexural modulus experimental and predicted values, (**b**) degree of adhesion *K*, and degree of dispersion *L* predicted values.

As shown in Figure 2a, the model predicted remarkably well the flexural modulus variation with *V_f_*. The maximum observed deviation between the model’s predicted values and the experimental ones was on the order of 3%. An increase in the filler-volume fraction led to an initial increase in the flexural modulus (Figure 2a). However, as *V_f_* approached the value of 14%, a subsequent decrease in the modulus with *V_f_* was observed. This is attributed to the creation of aggregates and a subsequent decrease in the effective filler-matrix contact area. The decrease in the effective filler-matrix contact area was also predicted by the PPM through the predicted degree of adhesion *K*, as shown in Figure 2b.

Finally, as *V_f_* increased, the predicted degree of dispersion, *L*, increased from 75 to 76%, i.e., it practically remained constant. As the glass spheres’ volume fraction, *V_f_*, increased, their dispersion within the hybrid polymer matrix became more and more difficult; however, due to the careful mixing of the components as well as the overall manufacturing conditions applied, it almost remained constant.

### 4.2. Glass Fibers of 0.2 mm in Length

**Figure 3:**
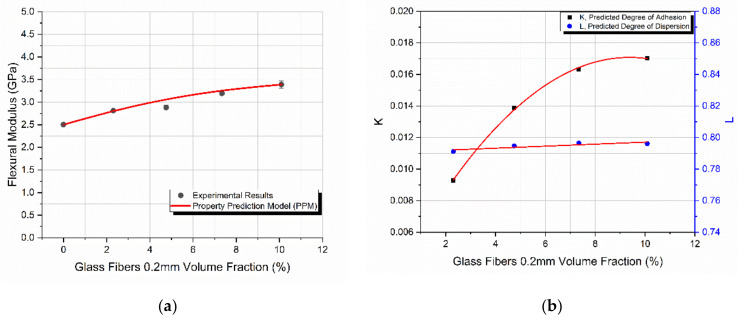
The starch-epoxy composites variation of 0.2 mm glass fibers with the filler-volume fraction *V_f_*, of (**a**) flexural modulus experimental and predicted values, (**b**) degree of adhesion *K*, and degree of dispersion *L* predicted values.

In the case of the 0.2 mm long fibers, the flexural modulus as a function of the filler-volume fraction is shown in Figure 3a. The behavior had the same trend as in the case of the 120 μm glass spheres. However, in this case the maximum filler-volume fraction, *V_f_*, achieved was 10%, corresponding to the maximum observed modulus as well, which was 3.4 GPa. Again, the model predicted remarkably well the modulus variation. The maximum deviation observed between the predicted values and experimental results was in all cases lower than 6.6%.

Similar to the glass spheres, where an initial increase and a subsequent decrease in flexural modulus was observed, in the case of the 0.2 mm glass fibers the initial increase in this behavior was only observed. This is because in this case, the maximum *V_f_* achieved was 10% due to the manufacturing limitations imposed by the amount of filler added beyond the maximum *V_f_*. It can be assumed that for *V_f_* values higher than 10%, a decrease in the modulus is expected to take place. This assumption is supported by the behavior of the predicted degree of adhesion, *K*, shown in Figure 3b. From this figure, it is clear that at 10% *V_f_*, where the composite material’s flexural modulus was at its maximum, *K* attained its maximum value as well, representing a threshold beyond which a subsequent decrease in modulus with *V_f_* is most likely to take place.

Again, as *V_f_* increased, the predicted degree of dispersion, *L*, remained practically constant. Similarly, as in the case of glass spheres, although as the filler content increased the dispersion of fillers within the matrix became more and more difficult due to the good mixing and overall manufacturing conditions applied, the degree of dispersion remained unaffected by the filler-volume fraction increase.

### 4.3. Glass Fibers of 3 mm in Length

**Figure 4:**
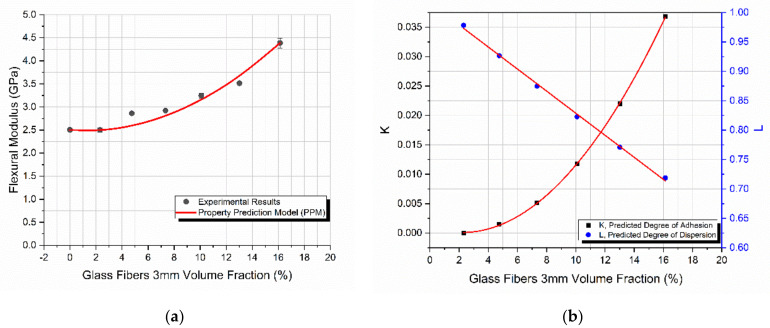
The starch-epoxy composites variation of 3 mm glass fibers with the filler-volume fraction *V_f_*, of (**a**) flexural modulus experimental and predicted values, (**b**) degree of adhesion *K*, and degree of dispersion *L* predicted values.

Figure 4a shows that an increase in the filler-volume fraction led to a continues increase in the composite flexural modulus. It should be mentioned that, because of the difficulties encountered in mixing the constituents, specimens with a filler-volume fraction, *V_f_*, higher than 16% were not manufactured. The PPM model predicted remarkably well the composites’ flexural behavior. The maximum observed deviation between the predicted values and experimental results was too low. The maximum achieved modulus was 4.4 GPa which corresponds to the maximum filler-volume fraction.

The increase in the flexural modulus is attributed to the increase in the effective filler-matrix contact area, which is also reflected in the PPM model’s predicted degree of adhesion, as seen in Figure 4b. Moreover, since the glass fibers introduced had a length of 3 mm while the average depth of the manufactured specimens was 2.5 mm, it can be assumed that as the *V_f_* increases, the more tightly packed glass fibers will be “forced” to become uni-directional rather than randomly oriented, resulting in a property increase, at least in the tested direction.

In the present case of 3 mm glass fibers, despite the good mixing and manufacturing conditions applied, the predicted degree of dispersion decreased with increasing *V_f_*. This is attributed to the length of the fibers which, as explained above, force the creation of agglomerations. The predicted degree of dispersion, *L*, ranged between 97% and 72%, which indicates an extremely good dispersion in the lower volume fractions and a fairly good dispersion even at high filler-volume fractions of the 3 mm glass fibers.

### 4.4. Glass Bubbles

**Figure 5:**
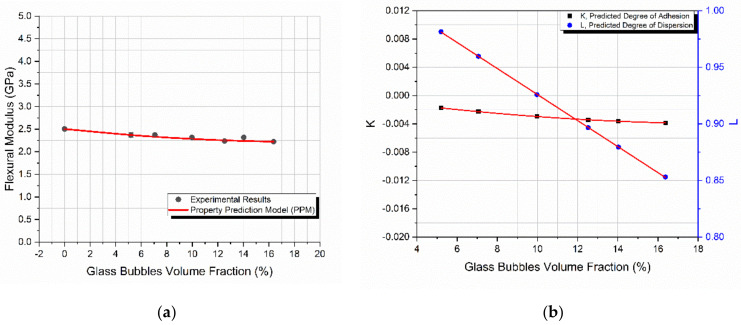
The starch-epoxy composites variation of glass bubbles with the filler-volume fraction *V_f_*, of (**a**) flexural modulus experimental and predicted values, (**b**) degree of adhesion *K*, and degree of dispersion *L* predicted values.

Figure 5a shows that an increase in the filler-volume fraction, *V_f_* leads to a continuous respective decrease in the flexural modulus. It should be mentioned that, because of manufacturing difficulties during mixing, specimens with a filler-volume fraction over 16.4% were not manufactured. Again, the PPM model predicted remarkably well the mechanical behavior. The maximum deviation of the model’s predicted value from the experimental ones was only 4.8%. The modulus variation observed with *V_f_* was expected, since the incorporation of glass bubbles into the hybrid matrix results in composites mainly useful in polymer weight reduction and insulation [19].

As for the *K* and *L* variation with *V_f_*, it is observed that *K* followed the same trend with the flexural modulus, while *L* again decreased with an increasing *V_f_*. In general, the increase or decrease in the modulus with the filler-volume fraction depends on the degrees of adhesion and dispersion as well as on the filler stiffness. In the case of glass bubbles, the filler stiffness was too low and on the order of 2.2 GPa, and thus no increase in the polymer composite system was expected to be observed as the filler-volume fraction increased.

### 4.5. Comparison of the Predicted and Experimental Results

**Figure 6:**
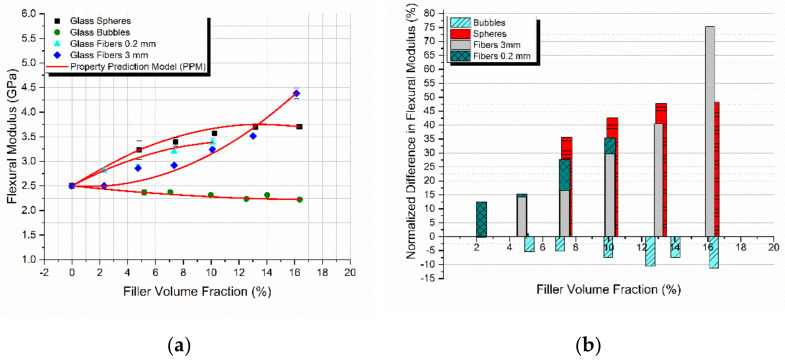
Comparison for all types of composites studied (**a**) between the experimental values and theoretical predictions as derived from the PPM model for the flexural modulus with the filler-volume fraction, and (**b**) the normalized modulus variation with *V_f_* relative to the matrix.

The flexural modulus of all four different composites manufactured is plotted as a function of the filler-volume fraction in Figure 6a. The PPM model was applied on the experimental results successfully and is also presented in the same figure.

As is evident, there was a constant increase in the flexural modulus for the composites reinforced with glass spheres and glass fibers. However, a small decrease was observed in the glass bubble-filled composites. The degradation of the mechanical properties of these composites confirms previous experimental results found in the literature [17], since these materials are used primarily for improving the dumping behavior of the material. The most notable observation yet in Figure 6a is that the PPM model perfectly predicted the behavior of the flexural modulus in all cases.

In Figure 6b the percentage variation of the flexural modulus as a function of the filler-volume fraction for all the different composites manufactured is presented. The highest increase in the modulus observed was on the order of 75% for the 3 mm glass fiber reinforced composites. The glass spheres and the 0.2 mm glass fiber reinforced composites followed with an increase of 48% and 35%, respectively. However, as mentioned above, the glass bubble reinforced composites underwent a flexural modulus decrease on the order of 11%.

**Figure 7:**
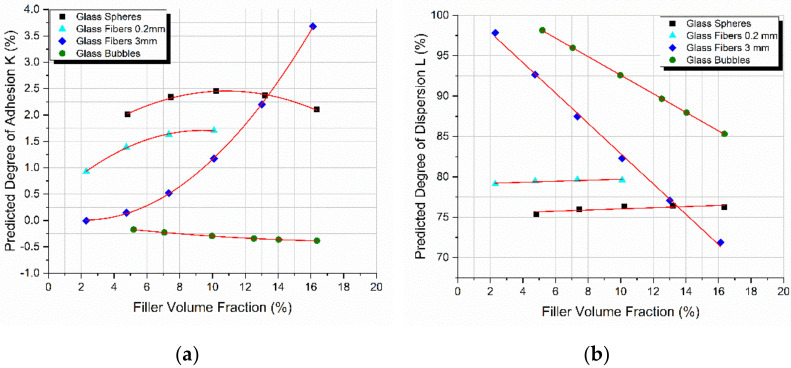
Predicted degree of (**a**) adhesion and (**b**) dispersion as a function of the filler-volume fraction for all types of composites studied.

As seen in Figure 7, the model predicted very low degrees of adhesion for all combinations of materials. Surprisingly, each of the four different fillers presented with a different degree of adhesion *K*, showcasing that adhesion depends on many different factors besides the type of the material, including the geometry and the size of the filler representing the load to strain transfer fraction from the matrix to the inclusion. The later fraction is related to the physical adhesion developed at the filler-matrix interface; however, this has a different value as compared to *K*. On the other hand, the degree of dispersion depending on the filler nature and dimensions either decreases and/or remains practically constant with an increase in *V_f_*.

**Figure 8:**
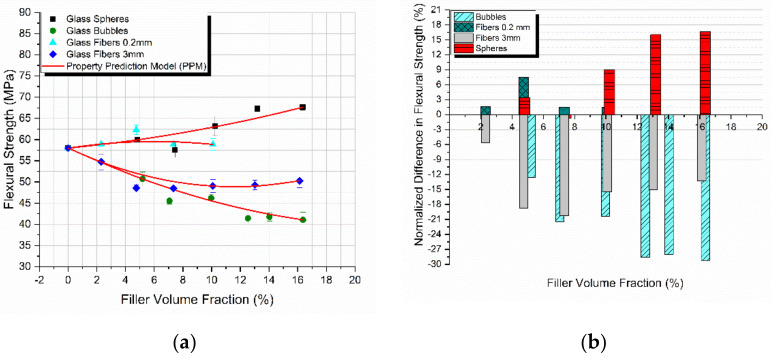
Comparison for all types of composites studied (**a**) between the experimental values and theoretical predictions as derived from the PPM model for the flexural strength with the filler-volume fraction, and (**b**) normalized strength variation with *V_f_* relative to the matrix.

The flexural strength of all four different composites manufactured is plotted as a function of the filler-volume fraction in Figure 8a. The PPM model was successfully applied on the experimental results and is also presented in the same figure. At this moment it should be emphasized, as already explained above, that parameters *K* and *L* are uniquely measured from modulus considerations alone. Despite the differences in variation between the flexural modulus and strength, again the PPM model perfectly predicted the flexural strength variation with *V_f_* in all cases, proving the adaptability of the PPM model in predicting various different properties [43].

In Figure 8b the percentage variation of the flexural strength as a function of the filler-volume fraction for all the different composites manufactured is presented. The highest increase observed was on the order of 16.5% for the glass spheres reinforced composites. A small increase of 7.5% in the flexural strength was observed in the case of the 0.2 mm glass fibers.

In general, well-bonded fillers (with a high *K*-value) give composites with a notably higher flexural strength while weakly bonded particles (with a low *K*-value) act as sources of “inherent” flaws provoking crack initiation leading to a decrease in flexural strength [44,45]. Indeed. in our case, as observed in Figure 7, materials characterized by high *K*-values (glass spheres, 0.2 mm glass fibers) exhibited an increase in flexural strength in the range of 7–16% at the maximum. On the contrary, materials showing low *K*-values (3 mm glass fibers, glass bubbles) exhibited a decrease in strength with the filler-volume fraction. Additionally, as observed in the case of the 3 mm glass fibers and at high filler-volume fractions where the *K*-values were high, a respective increase in strength with *V_f_* was observed at these *V_f_* values.

In Table 4 the flexural strength values presented in Figure 8 are supplemented with a statistical analysis of the results. A low standard deviation was found in all cases, indicating that the values tended to be close to the mean flexural strength values. Thus, the variation of the flexural strength values per case was very small and that is evident by the small coefficient of variation, with the largest being 5.76%.

### 4.6. SEM Micrographs

**Figure 9:**
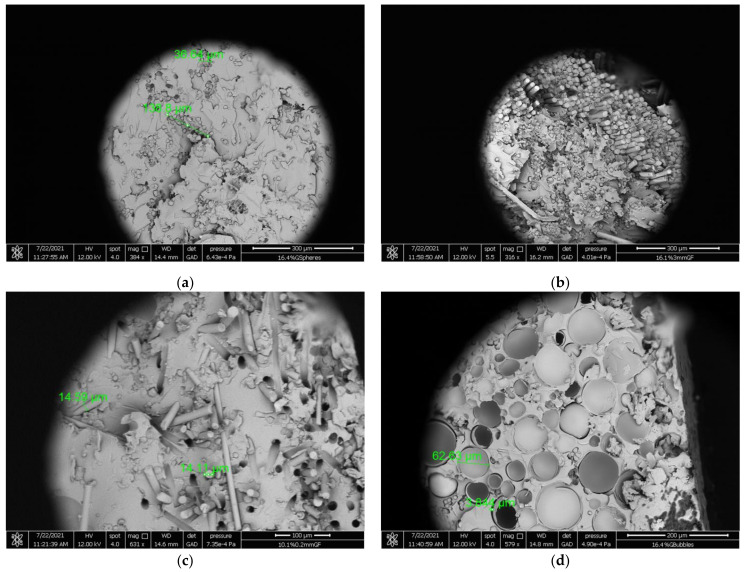
Scanning electron micrographs of the hybrid epoxy matrix composite specimens manufactured for (**a**) 26.4% *V_f_* glass spheres, (**b**) 31.6% *V_f_* 3 mm glass fibers, (**c**) 16.6% *V_f_* 0.2 mm glass fibers and (**d**) 32.5% *V_f_* glass bubbles.

In order to gain a better perspective and understanding of the manufactured specimens, scanning electron microscopy (SEM) photomicrographs of the fracture surface were taken and analyzed (Figure 9). In Figure 9a a SEM photomicrograph of a starch-epoxy/glass spheres composite for a 16.4% *V_f_* is shown. The aggregates that have formed can be observed, confirming our previous findings about the deterioration of the flexural modulus after a filler concentration threshold due to the agglomerations formed and the weak interfacial bond developed between the filler and the matrix.

Aggregates can also be observed in Figure 3b, where a SEM image of a starch-epoxy/3 mm glass fibers composite for a 16.1% *V_f_* is shown. However, the experimental mechanical results depicted a constant increase in the flexural modulus which was attributed to the possible uni-directional orientation of the tightly packed 3 mm glass fibers. This assumption is proven in Figure 9b, where uni-directional tightly packed glass fibers can be observed throughout the fracture surface area.

Next, in Figure 9c a SEM photomicrograph of a starch-epoxy/0.2 mm glass fiber composite for a 10.1% *V_f_* is shown. Although the aggregates are not as large as in the previous two cases, they are visible. However, the most striking observation is the empty holes in the resin, where the 0.2 mm glass fibers failed adhesively. This showcases the poor adhesion between the matrix and glass fibers predicted by the PPM model.

Finally, a SEM photomicrograph of a starch-epoxy/glass bubbles composite for a 16.4% *V_f_* is shown in Figure 9d. As can be seen, most of the glass bubbles are broken, leaving empty spaces and voids to develop within the composite structure. Thus, the modulus of the composite should remain almost constant and equal to that of the matrix, while due to the empty spaces existing in the matrix, a big decrease in strength with the filler-volume fraction is expected in this type of composite. Indeed, this is exactly the variation of the modulus and strength found and already presented in Figure 5a and Figure 8a, respectively.

To supplement the SEM results and the analysis of the flexural properties, the void content was estimated in each case. The void content of the specimens manufactured for the three-point bending tests was determined according to ASTM D 2734. The density of the specimens was calculated in accordance with ASTM D 1505. The void content appeared to be random in relation to the filler-volume fraction and for the different types of inclusions, which was also expected since the void content depends on several parameters, with one of the most important being the proper manufacturing process. However, in all specimens measured, it was found that the mean void content was on the order of 4% with a small variation depending on the filler geometry, reaching a maximum void content of 6.16% in the case of 4.8% *V_f_* in the glass spheres.

### 4.7. Geometrical Considerations

**Figure 10:**
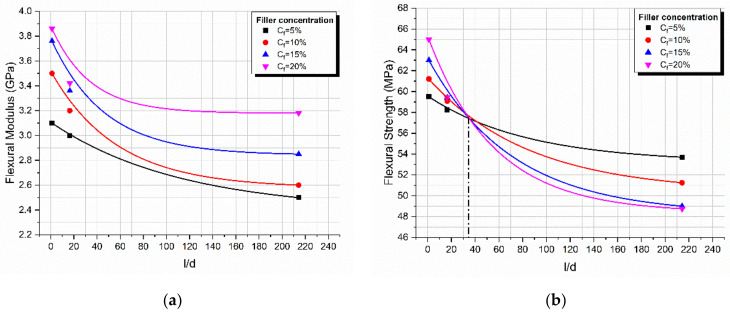
Variation with filler aspect ratio *l*/*d* for different filler loadings of (**a**) the flexural modulus and (**b**) the flexural strength.

**Figure 11:**
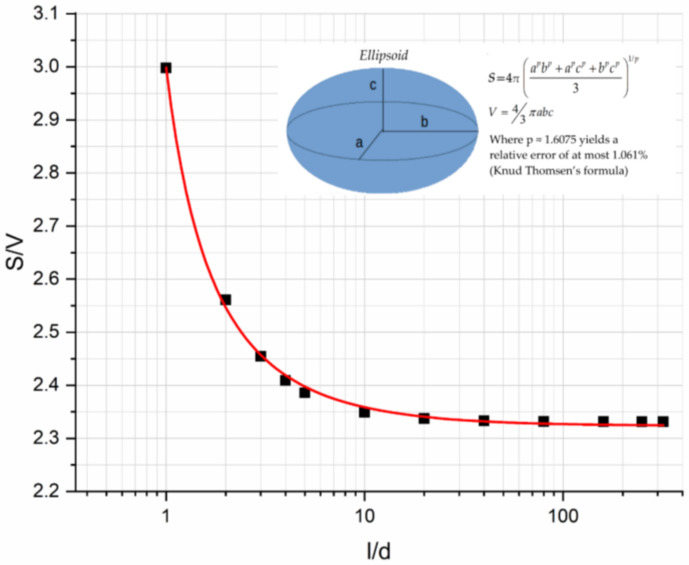
Ellipsoid inclusion area per unit volume as a function of the aspect ratio *l*/*d*.

Figure 10a shows the flexural modulus variation with the filler aspect ratio for glass spheres (*l*/*d* = 1), 0.2 mm glass fibers (*l*/*d* =16.42), and 3 mm glass fibers (*l*/*d* = 214.28) for different filler loadings. It can be seen that in all cases there was a continuous decrease in the modulus with *l*/*d*, while an increasing filler concentration with a respective increase in the modulus was observed due to the increase in the contact area. The decrease in the modulus with *l***/***d* is attributed to the decrease in the contact area per unit volume *S*/*V* of an ellipsoid inclusion as *l*/*d* increased, as shown in Figure 11.

Next, for the same composites, the flexural strength vs. the aspect ratio curve was plotted as shown in Figure 10b. As can be seen, a continuous decrease in the flexural strength with *l*/*d* was observed following the trend of the *S*/*V* vs. the *l*/*d* curve. However, the flexural strength depends upon the inherent flaws that exist on the inclusion surface; therefore, the rate of strength decrease is proportional to the filler loading. At low values of *l*/*d*, the strength increases with filler loading while as *l*/*d* increases, this behavior is inverted [46,47]. We also observed that for a critical value of *l*/*d* equal to 37.5, all curves passed through the same point (inversion point) corresponding to a flexural strength equal to 57 MPa approximately and this is independent on the inclusion type studied.

## 5. Conclusions

In the present investigation, four different types of glass filler reinforced starch-epoxy hybrid matrix composites were manufactured and tested in a three-point bending mode. More precisely, the fillers used in composites had different values in the aspect ratio *l*/*d* while the polymeric matrix was loaded with different amounts of filler particles. In addition, in all cases, the PPM model developed by the third author was applied for the prediction of the modulus and strength variation with the filler-volume fraction. The main results derived from the present investigation are as follows:

- A constant increase in the flexural modulus with the filler content for the composites reinforced with glass spheres and glass fibers was observed, while a small decrease in the glass bubble filled composites was found and this was in accordance with similar findings in the literature.
- A maximum increase in the modulus on the order of 75% was observed for the 3 mm glass fiber reinforced composites.
- In all cases, the PPM model perfectly predicted the flexural modulus variation with filler content.
- The degree of adhesion, *K*, as calculated from the PPM model followed the same trend of variation with filler loading as in the case of the flexural modulus.
- The degree of dispersion, *L*, as calculated from the PPM model, depending on the filler shape and dimensions and their tendency to agglomerate, in some cases decreased linearly while in other cases it remained practically constant.
- The maximum increase in the flexural strength observed was on the order of 16.5% for the glass sphere reinforced composites.
- In all cases, the PPM model perfectly predicted the flexural strength variation with the filler content.
- Materials characterized by high *K*-values (glass spheres, 0.2 mm glass fibers) showed an increase in flexural strength. On the contrary, materials with low *K*-values (3 mm glass fibers, glass bubbles) showed a decrease in strength with the filler-volume fraction.
- Concerning the effect of inclusion geometry on the flexural modulus, a decrease in the modulus with *l*/*d* was found and this was attributed to the decrease in the contact area per unit volume *S*/*V* of an ellipsoid inclusion as *l*/*d* increases.
- Finally, concerning the effect of inclusion geometry on the flexural strength, a decrease in strength with *l*/*d* was found, with a rate of strength decrease dependent upon the filler concentration.
- The flexural strength depends upon the inherent flaws that exist on the inclusion surface; therefore, the rate of strength decrease is proportional to the filler loading. At low values of *l*/*d*, the strength increases with filler loading while as *l*/*d* increases, this behavior is inverted.

## Figures and Tables

**Table 1:** Physical properties of corn starch.

Amylose Analogy (%)	16.9–21.3
Swelling power (g/g)	13.7–20.7
Solubility (%)	9.7–15
Water binding capacity (%)	82.1–97.7
Glass Transition Temperature (°C)	70

**Table 2:** Physical and geometrical characteristics of glass fillers [36,37,38,39].

Physical and Geometrical Characteristics	Glass Spheres	Glass Fibers 0.2 mm	Glass Fibers 3 mm	Glass Bubbles
Glass type	Soda-lime glass	E-Glass	E-Glass	Borosilicate glass
Cross section	Circular	Circular	Circular	Circular
Dimensions	^1^ diameter (av.): 120 μm	length (av.): 230 μm	length (av.): 3000 μm	diameter (av.): 65 μm
	^2^ *l*/*d*: 1	*l*/*d*: 16.42	*l*/*d*: 214.28	*l*/*d*: 1
Density	2.5 g/cm^3^	2.53–2.55 g/cm^3^	2.53–2.55 g/cm^3^	0.21 g/cm^3^
Bulk density	1.5 kg/dm^3^	0.45 kg/dm^3^	0.45 kg/dm^3^	0.125 kg/dm^3^
Softening temperature	470 °C	840 °C	840 °C	795 °C

^1^ av. denotes the 50th percentile of a normal distribution. ^2^ *l*/*d* represents length/depth and is a non-dimensional quantity.

**Table 3:** Glass filler-volume fractions.

Volume Fraction (%)			
Glass Spheres	Glass Fibers 0.2 mm	Glass Fibers 3 mm	Glass Bubbles
4.82	2.31	2.31	5.20
7.45	4.75	4.75	7.00
10.23	7.34	7.34	9.97
13.19	10.09	10.09	12.53
16.35	-	13.01	14.04
-	-	16.13	16.37

**Table 4:** Statistical analysis of the flexural strength values.

Glass Type	Filler-Volume Fraction (%)	Flexural Strength (MPa)	Standard Deviation (MPa)	Coeff. of Variation (%)
Hybrid Resin	0.00	57.98	0.26	0.45
Glass spheres	4.82	59.94	0.50	0.83
	7.45	57.57	1.77	3.08
	10.23	63.21	2.23	3.52
	13.19	67.28	0.70	1.03
	16.35	67.62	0.61	0.91
Glass fibers 0.2 mm	2.31	58.95	0.54	0.92
	4.75	62.35	1.13	1.81
	7.34	58.85	1.77	3.00
	10.09	58.87	1.35	2.30
Glass fibers 3 mm	2.31	54.71	1.85	3.37
	4.75	48.55	0.74	1.53
	7.34	48.47	2.79	5.76
	10.09	49.04	1.52	3.09
	13.01	49.29	1.13	2.30
	16.13	50.27	1.65	3.27
Glass Bubbles	5.20	50.69	1.63	3.21
	7.06	45.55	0.79	1.73
	9.97	46.17	0.83	1.81
	12.53	41.43	1.52	3.66
	14.04	41.75	0.98	2.36
	16.37	41.05	1.82	4.44

## Data Availability

The data presented in this study are available in manuscript.
